# Bacteriological load in Holstein Friesian cows with dystocia

**DOI:** 10.3389/fvets.2024.1456324

**Published:** 2024-12-11

**Authors:** Florin Nechifor, Ștefan Gregore Ciornei, Petru Roșca

**Affiliations:** Department Clinics, Reproduction, Faculty of Veterinary Medicine, University of Life Sciences (IULS), Iași, Romania

**Keywords:** cow, dystocia, eutocic, bacteria, puerperium, fertility

## Abstract

The research took place on a farm in North-Eastern Romania with Holstein Friesian cows aged between 3 and 9 years. Bacteriological investigations were carried out throughout the year 2023, on a total of 35 cows, including 25 multiparous cows and 10 primiparous cows, 23 cows had eutocic parturitions and 12 cows had dystocic parturitions, during the first 3 weeks postpartum. In the case of dystocic parturition, biological samples yielded isolates including 9.3% strains classified as *Staphylococcus*, 8.1% strains of *Escherichia coli*, 4.1% strains of *Arcanobacterium*, and 2.3% strains of *Klebsiella*. Other bacterial types were identified in lesser proportions. In the case of eutocic parturition, *Escherichia coli* was most frequently isolated: 18.6% of the isolated bacterial strains, followed by 5.8% *Pseudomonas* spp., 4.6% *Enterococcus* spp., 4.6% *Streptococcus* species, 3.5% *Staphylococcus* spp., and *Corynebacterium* spp., and 2.3% *Arcanobacterium* spp. It is noted that on the 7th day of puerperium, the mean value of colony-forming units per milliliter (CFU/mL) was 74 × 10^4^ CFU/mL in normal calving cows compared to 29 × 10^6^ CFU/mL in cows with dystocia. The total number of recorded germs increases significantly during the first 14 days postpartum in all cases, higher levels being shown in cows with dystocic calving. For statistical analysis, the independent t-test (*p* < 0.05) was made by using the SPSS 16 software. The object of the research is the bacteriological load in Holstein Friesian cows depending on the type of eutocic parturition/dystocia.

## 1 Introduction

Puerperal infections of the reproductive tract in dairy cows can lead to fertility problems and represent a relevant economic problem due to their frequency. Uterine disorders lead to the reduction in cow welfare due to inflammation of the genital tract and they should not be underestimated (1, 2).

Understanding the morpho-functional activities of the genital apparatus is an essential condition for specialists who prevent and treat disorders that may occur at this level (3).

The general understanding is that the reproductive ability of animals, expressed by the term fertility, is based on two anatomo-physiological coordinates of the genital apparatus: the structural component and the dynamic component, represented by the peculiarities of reproductive function (4, 5). These two values are integrated into a morphophysiological complex that constitutes the entire animal organism (6, 7).

Through gynecological examinations and by clinical and laboratory research, the causal agents of reproductive disorders have been highlighted. These agents can lead to various lesion and functional changes of the genital apparatus (8, 11).

The postpartum period is predominantly included in the complex of conditions affecting sexual activity (9, 10, 12).

The puerperal period is conditioned and influenced by several factors: the physical and health status of the female, the course of parturition, the quality of parturition assistance, nutrition, milk production, environmental conditions, or stress factors (13, 16, 17).

Under these conditions, this period is characterized by: postpartum uterine involution, lochia elimination, postpartum ovarian activity resumption and postpartum uterine bacteriology (14, 18).

The various obstetric disorders that can occur during this period cause serious disturbances in the course of the puerperium, negatively influencing the main reproductive parameters, including fecundity and female fertility (7, 15).

However, bacterial contamination of the uterus in postpartum cows is common and the development of uterine inflammation depends on local immunity and the intensity of contamination (6).

Obstetric disorders that may occur during this period can cause serious disturbances in the course of the puerperium.

Infections in the puerperal period may lead to puerperal metritis or clinical endometritis. Thus, uterine bacterial infections have been shown to adversely affect uterine and ovarian function and fertility (13). These conditions can be so severe that they can permanently compromise reproductive function (17).

In addition to understanding the physiological phenomena that occur during the puerperal period, it is recognized that within the reproductive cyclogram, in case of disturbances that may occur, some modifications in the female’s subsequent fertility are observed (9, 15).

The numerous functional transformations that take place during the postpartum period, recorded at the cellular level up to the level of organs and systems, can have different effects negatively influencing the body’s recovery and delaying or blocking the resumption of sexual function (18).

Under the influence of these factors and in the conditions of the great lability of postpartum body recovery processes, the pathology of the puerperal period is diverse and complex, being directly related to the pathology of parturition and the periparturient period (16).

Monitoring the puerperal period involves coordinating the factors that determine the smooth running of sexual processes, with an important component being the optimal resumption of physiological and morphofunctional parameters close to the ante-partum period (14).

The object of the research is to determine the bacteriological load in Holstein Friesian cows depending on the type of eutocic parturition/dystocia.

## 2 Materials and methods

Bacteriological investigations were carried out throughout the year 2023, on a total of 35 cows, including 25 multiparous cows and 10 primiparous cows, 23 cows had eutocic parturitions and 12 cows had dystocic parturitions, during the first 3 weeks postpartum. The research took place in North-eastern Romania on Holstein Friesian cows aged between 3 and 9 years.

Sampling was performed with an exudate swab made in the laboratory. For this, 40 cm metal rods were used, the ends of which were protected with cotton wool. One of them was inserted into a 30 cm test tube and finally they were distributed individually in bags to be sterilized by autoclaving. Sterilization was carried out at 121°C for 60 min. In this way, a proper sterilization and a suitable size for this type of harvest was ensured.

For the isolation and identification of potentially pathogenic bacterial agents, both standard culture media and differential and selective media were used.

Differential media contain substrates for specific bacterial enzymes or cytotoxins and an indicator that confirms the reactivity on this substrate. This category also includes blood agar, which differentiates between hemolytic and non-hemolytic bacteria, or lactose media with various pH indicators. Selective culture media for bacteria contain specific indicators that confirm certain characteristics of bacteria, such as their ability to use certain substrates, to produce certain enzymes, or to resist certain antimicrobial agents.

The selective and differential media used were: EMB Agar (Oxoid); Mac Conkey Agar (Merck); Chapman Agar (Oxoid); Columbia Agar (Oxoid); Infusion Brain Heart Agar (Oxoid); Agar-bile-esculin-sodiumazide; Brucella Agar (Oxoid); CCDAgar (Charcoal Cefoperozone Deoxycholate agar, Oxoid); SPS Agar (Sulphite Polymyxin Sulfadiazine, Oxoid); TSI (triple sugar iron agar, Oxoid); MIU (motility, indole, urease, Oxoid); MILF (motility, indole, lysinedecarboxylase, phenylalaninedeaminase, Oxoid); Simmons Agar (Oxoid) and oxidase test.

Pathological materials were subjected to laboratory investigations according to the general guidelines of bacteriological diagnosis.

The culture media used are produced by: Oxoid (UK) and Merg (Germany).

Selective media contain substances that inhibit the growth of contaminating bacteria, possibly collected during sampling. All special culture media, used for bacterial isolation, contain, according to the technical file, different substances that can inhibit or favor the multiplication of certain species of microorganisms. Depending on their recommendation, their composition is different.

For identification of bacterial strains Api galleries were used.

Api systems allow easy and rapid identification of 20–30 biochemical tests of aerobic and anaerobic bacteria.

The API tests were selected according to the results obtained following the examination of the tinctorial, morphological and cultural characters of the isolated strains.

API 20 A galleries were used to test anaerobic bacteria.

The evaluation methodology is a classic one in microbiology. After the isolation of the strains in pure culture, the bacteria were subjected to specific work steps for biochemical testing using the API galleries. The protocol is extensive and we are not allowed to describe the work steps, but summarizing we can mention: isolation in pure culture on the solid medium; obtaining a bacterial suspension equivalent to 0.5 Mc Farland (1.5×10^8^ CFU), adding it to the wells of the API galleries. Incubation at 37°C for 24–48 h under aerobic or anaerobic conditions, checking each test based on color change by automatic reading with the help of the API reading system.

Determinations were made using the API LAB Plus.

The complex biochemical identification systems used were: mini API galleries for API 20 NE, API 20 E, API 32 GN. ID32GN is used for the identification of Gram-negative species, but we used API 32 E, which also allowed the identification of other non-Enterobacteriaceae species. The galleries used are produced by Biomerieux. To reconstitute the substrate, the galleries were inoculated with a bacterial suspension made from the studied colony. Bacterial suspensions were made from the 24-h strains brought to a bacterial density equivalent to the 0.5 Mc Farland standard (1.5×10^8^ cfu).

When the results were inconclusive after the first reading, the galleries were further incubated for another 24 h. After reading, additional agents were introduced, which differ from one identification system to another: Api 20E (TDA, IND). Sometimes, the substrates in the wells of the API galleries are not conclusive enough in terms of color shift and the reading can give errors. In this case, it is useful and in practice it is recommended that the API galleries be maintained for another 24 h in the thermostat and only then the final reading is done.

The bacteriological examination protocol requires isolating bacteria from pathological materials on culture media and identifying the cultures.

Inoculation was performed directly onto culture media without prior processing. The inoculum was spread using an exudate swab or an inoculation loop onto agar medium. Inoculum spreading was performed using the quadrant streaking technique to obtain isolated colonies.

Inoculation on liquid media was carried out using a Pasteur pipette, transferring the biological sample from the transport tube into the appropriate growth medium.

Following inoculation, culture media were incubated in a thermostat at 37°C under aerobic and anaerobic conditions.

The pathological material taken from the patient was discharged into one milliliter of sterile physiological serum, from which decimal dilutions were then carried out up to dilution 10^8^. From each dilution, 1 mL was taken and distributed in two sterile Petri dishes, over which the Mueller Hinton Agar medium was added, melted and cooled to 45°C so as not to influence cell viability in the suspension, and the dishes were incubation at 37°C. After 24 h, bacterial colonies were counted from the last plates, where the density of the colonies allowed them to be counted. The results obtained on the two dilution plates were averaged and multiplied by the dilution factor, thus obtaining the total number of germs per 1 mL of biological sample suspension. The results were reported in CFU/mL.

The results of the bacterioscopic examination conducted on the collected pathological materials revealed bacterial morphologies and different staining affinities, depending on the sampling area and the evolution of the infectious site. The purpose of the bacterioscopic examination was to guide bacteriological investigations to obtain relevant results.

Microscopic examination of Gram-stained smears identified fields with: coccal forms of 1 μm, arranged in shorter or longer chains, or grouped in clusters, stained Gram-positive; bacilli with club-shaped or rounded ends and deformed bacterial bodies with endospores, Gram-positive, of various sizes, some unbranched, non-sporulated, grouped in palisades, Chinese letter shapes, or short chains, with or without metachromatic granules; branched, filamentous filaments, long bacilli, some fusiform, Gram-positive or Gram-negative, some arranged radially; kidney-shaped cocci, arranged in diplo or tetrads, stained Gram-negative; cocobacilli or Gram-negative bacilli, with or without bipolar staining, sporulated or non-sporulated.

### 2.1 Statistical analysis

For statistical analysis, the independent *t*-test (*p* < 0.05) was made by using the SPSS 16 software.

## 3 Results

### 3.1 Bacteriological examination from a quantitative point of view

**Figure 1:**
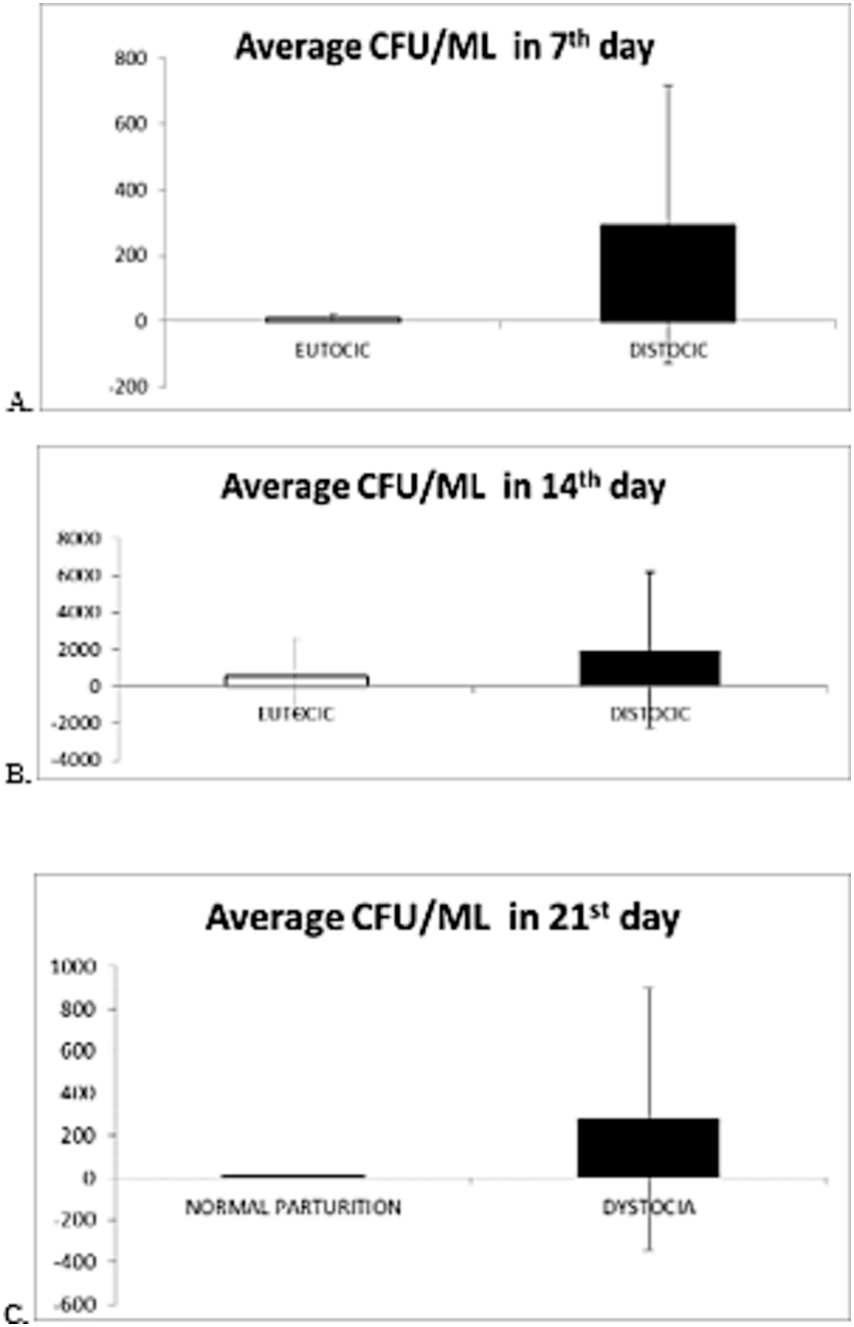
Graphical representation of the average value and standard deviation of evolution of the microbial load from cows in 7th day **(A)**, 14th **(B)**, 21st day **(C)** of puerperium with normal parturition/dystocia.

It is observed that, in the 7th day of puerperium the average value of colony-forming units per milliliter (CFU/mL) was 74 × 10^4^ CFU/mL in cows with normal parturition compared with 29 × 10^6^ CFU/mL in cows with dystocia. In this situation, the differences in the bacterial load are obvious (Figure 1A).

For *p* < 0.05 (independent student t-test), the difference between average CFU/mL of 7th day samples from cows with normal parturition and average CFU/mL of 7th day samples from cows with dystocia is statistically significant.

At 14th day of puerperium the average value for cows with normal parturition was 52×10^6^ CFU/mL and for those with dystocia the average grew up until 19×10^7^ CFU/mL (Figure 1B).

For *p* < 0.05 (independent student t-test), the difference between average CFU/mL of 14th day samples from cows with normal parturition and average CFU/mL of 7th day samples from cows with dystocia is not statistically significant.

At 21st day of puerperium, the average CFU/mL for cows with normal parturition was 27×10 CFU/mL, while for those with dystocia the average was 27×10^6^ CFU/mL (Figure 1C).

For *p* < 0.05 (independent student t-test), the difference between average CFU/mL of 14th day samples from cows with normal parturition and average CFU/mL of 7th day samples from cows with dystocia is statistically significant.

The results obtained from the quantitative evaluation of biological samples do not indicate significant differences in cows with eutocic calving concerning CFU/mL, which are consistent with those mentioned in specialized literature. For most cows with eutocic calving, the microbial load varied at 7 days between 10^3^–10^7^, at 14 days between 10^5^–10^6^, and at 21 days between 10^4^–10^5^. Similarly, there are no major differences between the compared results obtained in the study for cows with dystocic calving, except for one case, where the bacterial load varied within wider limits. Thus, in cow with identification number 4092, with dystocic calving, the total number of germs varied from 25×10^5^ at 7 days postpartum, to 11×10^8^ at 14 days, and slightly decreased to 16×10^7^ at 21 days. Bacteriological examination of vaginal and cervical biological samples from cows in the postpartum period led to the isolation of numerous bacterial strains.

The primary analysis of these data shows that, from these biological samples, 9 (5.24%) bacterial strains were isolated in pure cultures and 163 (94.76%) mixed bacterial strains (Table 1).

**Table 1:** Classification of bacterial strains according to respiratory type and tinctorial affinity.

Species		Total number of bacterial strains	Respiratory type	Strains		Gram +		Gram –	
				Nr.	%	Nr.	%	Nr.	%
Cows		172	Aerob bacteria	163	94.76	71	41.2	92	53.4
Parturition Eutocic 23 (65.7%)	Parturition Distocic 12 (34.3)		Anaerob bacteria	9	5.24	4	0.23	5	0.29
Total				172	100%	75	43.6	97	56.4

The classification of the isolated microorganisms according to respiratory type revealed the predominant presence of 163 aerobic bacterial strains, of which 71 were Gram-positive and 92 Gram-negative. Anaerobic bacteria were present in small numbers, with 9 (5.24%) strains, of which 4 Gram-positive and 5 Gram-negative strains.

By correlating cultural, morphological, and biochemical characteristics, the majority of isolated bacterial strains were classified up to the level of bacterial species.

Microbiological investigations revealed that *Escherichia coli* was the most frequently isolated bacterium from vaginal and cervical samples, with 46 (26.7%) strains, followed by *Staphylococcus* spp. with 22 (12.8%) strains and *Arcanobacterium pyogenes* with 11 (6.4%) strains (Table 2).

**Table 2:** Classification of bacterial strains according to the type of parturition.

Bacterian strains			Eutocic parturition		Distocic parturition	
			Nr.	%	Nr.	%
Gram +	*aerob*	*Staphylococcus*	6	3.5	16	9.3
	*aerob*	*Streptococcus*	8	4.6	-	
	*aerob*	*Enterococcus*	8	4.6	-	
	*aerob*	*Micrococcus*	7	4.1	-	
	*aerob*	*Corynebacterium*	6	3.5	3	1.7
	*aerob*	*Arcanobacterium*	4	2.3	7	4.1
	*aerob*	*Listeria*	1	0.5	2	1.1
	*anaerob*	*Clostridium*	5	2.9	2	1.1
Gram –	*aerob*	*Escherichia coli*	32	18.6	14	8.1
	*aerob*	*Citrobacter*	2	1.1	3	1.7
	*aerob*	*Pseudomonas*	10	5.8	-	-
	*aerob*	*Klebsiella*	4	2.3	4	2.3
	*aerob*	*Proteus*	4	2.3	2	1.1
	*aerob*	*Pasteurella*	3	1.7	-	-
	*aerob*	*Actinomyces*	3	1.7	1	0.5
	*aerob*	*Enterobacter*	2	1.1	3	1.7
	*aerob*	*Serratia*	-	-	1	0.5
	*anaerob*	*Bacteroides*	-	-	1	0.5
	*aerob*	*Neisseria*	7	4.1	-	-
	*aerob*	*Actinobacillus*	-	-	1	0.5

From the data obtained in the bacteriological examination, it arises that, based on the course of parturition, out of the 35 cows, 23 experienced eutocic calving from which 110 bacterial strains were isolated, while dystocic calving was observed in 12 cows from which 62 bacterial strains were isolated. Additionally, it is noted that *E. coli* was predominantly isolated from the biological samples, both in the case of eutocic calving (19.14%) and dystocic calving (9.21%).

In the case of dystocic birth, 9.3% of the strains were classified in the *Staphylococcus* genus, 8.1% *E. coli* strain*s*, 4.1% *Arcanobacterium* strains, and 2.3% *Klebsiella* strains isolated from the biological samples. In the case of eutocic calving, *E. coli* was the most frequently isolated: 18.6% of the bacterial strains isolated, followed by 5.8% *Pseudomonas* spp.

### 3.2 Bacteriological examination from a qualitative point of view

The results of the bacterioscopic examination, performed on the pathologic specimens, revealed different bacterial morphologies and dye affinity, depending on the area of sampling and the evolution of the infectious outbreak. The aim of the bacterioscopic examination was to guide the bacteriologic investigations in order to obtain appropriate results.

**Figure 2:**
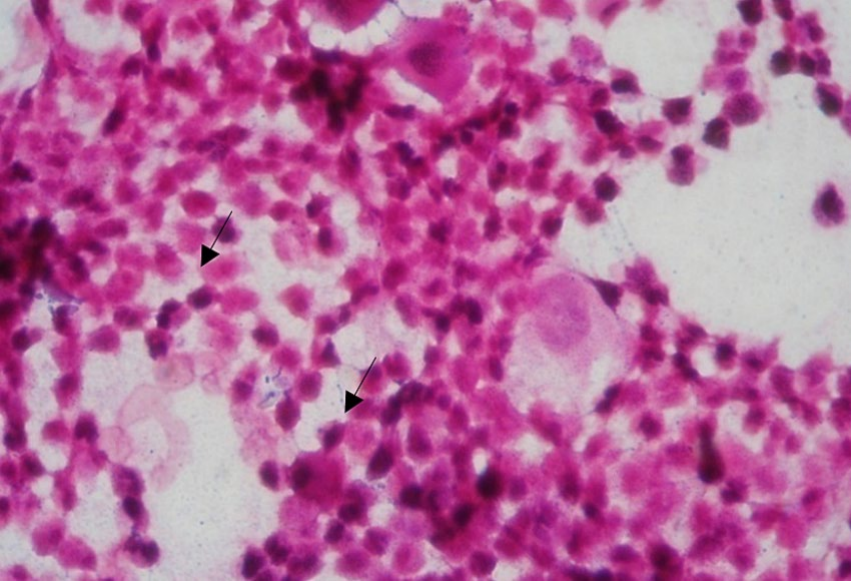
The microscopic aspect of the cow’s genital secretion 21 days post-partum. Microscopic field with different bacteria types. Direct smear, Gram coloration, 20×100.

Microscopic examination of Gram-stained smears identified fields with:

- 1 μm cocoid forms, arranged in shorter, longer chains or grouped in clusters, Gram stained Gram positive;
- Rod-shaped or rounded bacilli with deformed bacterial bodies of endospores, Gram-positive, of various sizes, some unamamified, unsporulated, grouped in palisades, Chinese idiograms or short chains, with or without metachromatic granulation;
- Branched filaments, filamentous, long bacilli, some fusiform, Gram-positive or Gram-negative, some radially arranged;
- Coci reniform, arranged in tetrads, Gram-negative stained;
- Cocobacilli or Gram-negative bacilli, polymorphous, with or without bipolar coloration, sporulating or non-sporulating (Figure 2).

## 4 Discussion

Sampling is an important step for bacteriological examination. For the bacteriological examination, sterile genital secretions were collected from the cervix and vagina.

The direct bacterioscopic examination often does not provide accurate identification of the bacteria. Therefore, the working protocol of the bacteriological examination involves isolating bacteria from pathological materials on culture media and identifying the cultures themselves.

To achieve this, pathological materials were inoculated onto both regular aerobic, anaerobic culture media, as well as special and selective media. Most samples were investigated within 30 min of collection.

Puerperal infections of the genital tract in cows can lead to a series of fertility problems and due to their incidence, they are still an essential problem from an economic point of view (5, 20). Also, postpartum hygiene of cows has a major influence on the course of puerperal inflammation and recovery of sick cows (18).

Ante- and post-partum hygiene has a major influence on the development of puerperal disorders (16).

According to the authors, this bacterial contamination is not specific, but includes a large number of different bacteria. Sheldon et al. (10), attempted to classify bacteria isolated from the postpartum uterus based on their pathogenic potential and assigned high pathogenicity to *Trueperella pyogenes*, *Prevotella* spp.*, E. coli* and *Fusobacterium* spp.

The biological barriers are broken and thus the bacteria ascend to the vagina and uterus. This is an inevitable consequence, but it seems that some cattle face more challenges in controlling this bacterial contamination than other cows (1, 18).

According to the specialized authors, this bacterial contamination in this species is not specific, but includes an extremely large number of different bacteria. Thus, there is a risk that the bacterial contamination becomes bacterial infections, because the cow’s defense mechanisms do not cope (1). Sheldon et al. (10), states that in the first 14 days post-partum, a wide variety of bacteria can be detected in about 90% of cattle.

According data, Williams et al. (7), reached conclusion; *E. coli* and *Fusobacterium necrophorum* were associated with endometrial inflammation, while the presence of gram-negative staphylococci and streptococci seemed to exert a protective effect against the condition after postpartum calving.

This was true for *bacteroides* spp. *Escherichia coli*, for example. These bacteria were also detected significantly more often in the sick animals compared to the healthy ones (6, 21).

Moore et al. (17), also reported that coagulase negative Staphylococci were more prevalent in animals that did not show uterine infections and reduced the risk of abnormal vaginal discharge.

The genus *Actinobacillus* spp. was isolated from the postpartum uterus of dairy cows, but has not been associated with positive or negative effects on uterine health, only with epididymitis in rams (12).

Acinetobacter spp. is described by Sheldon et al. (4) as a potential pathogen of uterine infections.

*Staphylococcus* spp. in our research and the increased occurrence of potential pathogens genera, as seen with *Clostridium* spp. in cows with dystocic parturitions versus those with eutocic parturitions.

Uterine bacteriology and defense capacity are important in the evolution of the puerperal period, various studies report that the bacteria most commonly associated with uterine infections include *E. coli, Corynebacterium, Actinomyces, Arcanobacterium pyogenes*, and anaerobic bacteria such as *Fusobacterium necrophorum, F. nucleatum and Bacteroides* spp. (18, 19), compared to similar results obtained by us.

Other recent research shows that *E. coli* and *Staphylococcus* spp. were the most frequently isolated bacteria, a total of 52 bacterial isolates (20).

Even in the case of subclinical endometritis reported by Sikra et al. (21), uterine bacteriology shows an included Gram-positive bacteria in percentage of 62.5 and 37.5% Gram-negative bacteria. The highest incidence was reported for *Escherichia coli* (16.66%), vancomycin-resistant *Enterococcus* spp. (16.66%), *Staphylococcus* spp. (14.58%) and *Streptococcus agalactiae* (12.5%).

## 5 Conclusion

Postpartum bacterial load in the uterus first increased and then decreased in cows with dystocic parturition; bacterial diversity in the uterus of cows with eutocic parturitions was lower. Characteristic changes in the relative abundance of uterine bacteria in cows with dystocia included increased *Staphylococcus* and *Corynebacterium*, decreased *Actinomyces* and *Bacteroides*.

## Data Availability

The raw data supporting the conclusions of this article will be made available by the authors, without undue reservation.
